# The DECIDE Cattle Barometer: a cross-country monitoring and decision support tool for respiratory pathogens

**DOI:** 10.1186/s13620-026-00356-0

**Published:** 2026-07-30

**Authors:** Jade Bokma, Charlotte Doidge, Stan Jourquin, Miel Hostens, Saba Noor, Inge Santman-Berends, Stefaan Ribbens, Julien Evrard, Sebastiaan Theuns, Cosme Sánchez-Miguel, Gerdien van Schaik, Jasmeet Kaler, Bart Pardon

**Affiliations:** 1 Department of Internal Medicine, Reproduction, and Population Medicine, Faculty of Veterinary Medicine, Ghent University, Merelbeke, Belgium; 2 Department of Animal Sciences and Aquatic Ecology, Faculty of Bioscience Engineering, Ghent University, Ghent, Belgium; 3 School of Veterinary Medicine and Science, University of Nottingham, Sutton Bonington, LE12 5RD UK; 4 Department of Farm Animal Health, Faculty of Veterinary Medicine, Utrecht University, Utrecht, The Netherlands; 5 Department of Research & Development, Royal GD, AA Deventer, The Netherlands; 6 Animal Health Service Flanders (DGZ-Vlaanderen), Torhout, Belgium; 7 Regional Association for Animal Identification and Health (ARSIA) asbl, Ciney, Belgium; 8 PathoSense BV, Ghent, Belgium; 9 Department of Agriculture, Food and the Marine, Cork Regional Veterinary Laboratory, Cork, Ireland; 10 Department of Animal Science, College of Agriculture and Life Sciences, Cornell University, Ithaca, NY USA; 11 Faculty of Bioscience Engineering, Department of Animal Science and Aquatic Ecology, Ghent University, Ghent, Merelbeke 9000 Belgium

**Keywords:** Early warning, Calves, Decision-making, Interactive dashboard, Data management, Diagnostic methods, Respiratory tract infections

## Abstract

**Background:**

Bovine respiratory disease (BRD) remains one of the most impactful health challenges in cattle, driving antimicrobial use, economic losses, and welfare concerns. Effective control requires insight into circulating pathogens. However, differences in sampling strategies, diagnostic methods, and data formats hinder cross-country interpretation. During a European stakeholder consultation, farmers called for simple tools that clearly communicate trends in pathogen occurence, while veterinarians stressed the need for data-driven dashboards that turn complex data into practical insights to support evidence-based advice and farmer–veterinarian collaboration. To address these priorities, we aimed to develop and evaluate the Cattle Barometer: an interactive, cross-country dashboard integrating laboratory-confirmed BRD pathogen data from multiple European diagnostic laboratories.

**Methods:**

Diagnostic laboratories in Europe provided anonymised test results from cattle with respiratory disease. Data included identified pathogens, sample type, diagnostic method, date, and location proxies. Heterogeneous datasets were harmonised using scripted pipelines, including cleaning, recoding, anonymisation, and transformation into standardised formats. Farm-level monthly pathogen positivity was calculated and incorporated into a dashboard. Usability of the first prototype was evaluated through a survey (*n* = 25), including a System Usability Scale (SUS) assessment, open-ended questions, and task-based testing among veterinarians, researchers, laboratory scientists, advisors, and farmers.

**Results:**

Fourteen datasets from six laboratories, representing over 8000 farms sampled between 2016 and 2024, were integrated into one dataset. The dashboard enables interactive analysis of trends in proportion of positive samples by time, location, production type, and diagnostic methodology.

The survey showed the tool to be promising, but still requiring refinement to achieve optimal user-friendliness (SUS score 60, 60% successfully completed the assigned task). Usability feedback highlighted strengths in data accessibility, but identified opportunities for improvement in navigation, visual load, date filtering, and interface scaling. Most respondents indicated they would use the tool in the future, and the majority were comfortable with the tool being available for veterinarians (88%) and farmers (72%).

**Conclusions:**

The Cattle Barometer provides an open-source framework for BRD monitoring and decision-making across Europe, with potential extension to other species and diseases. Future development should focus on automation to enable continuous data streams with daily updates and thereby enhance its surveillance value.

## Background

Bovine respiratory disease (BRD) is one of the most impactful diseases in cattle, contributing to substantial antimicrobial use, major economic losses, and compromised animal welfare [1–3]. Next to calf-factors and management practices, multiple viruses (e.g. bovine respiratory syncytial virus, parainfluenza type 3) and bacterial agents, such as *Mycoplasma bovis (*also *Mycoplasmopsis bovis)* and members of the *Pasteurellaceae* family are associated with BRD [4–6]. The prevalence and relative importance of these pathogens vary over time and between regions [3]. Consequently, timely awareness of circulating respiratory pathogens is increasingly recognised as an essential component of evidence-based disease prevention and control, supporting targeted vaccination strategies, biosecurity measures, purchase decisions, and prudent antimicrobial use [3, 7–9].

Several surveillance initiatives have been developed to monitor respiratory pathogens in cattle and to provide insight into national or regional pathogen occurrence [6–8]. These systems represent valuable resources for veterinary practitioners and policymakers, but they generally operate within individual countries or diagnostic networks. Consequently, they do not account for the transboundary nature of cattle production and pathogen circulation across Europe. Next to this, meaningful comparison of diagnostic data between countries is often complicated by differences in sampling strategies (e.g., nasal swabs versus broncho-alveolar lavage) [10], diagnostic methods [11], laboratory protocols [12], and reporting practices (e.g. Ct values versus semi-quantitative results) [11, 13, 14]. These challenges highlight the need for harmonised approaches that integrate heterogeneous diagnostic data while providing sufficient context for accurate interpretation.

Nevertheless, the successful implementation of monitoring and decision-support tools depends not only on technical performance but also on their acceptance and usability by end users [7, 15]. Prior to the present study, stakeholder requirements were explored through multinational focus groups involving cattle farmers from six European countries and an online qualitative survey among British and Irish cattle veterinarians [16, 17]. These studies show that farmers value user-friendly technologies that improve their understanding of herd health, support reviewing historical data, and enhance self-reliance [17]. Veterinarians emphasise data-driven tools that translate complex datasets into actionable insights, strengthening evidence-based advice and farmer–veterinarian interaction [16]. However, concerns remain about data quality, transparency, usability, information overload, and data sharing, highlighting the need for tools that support effective communication and collaboration [16, 17]. These findings informed the design principles of the tool presented here.

The DECIDE project (Horizon 2020, No. 101000494) aimed to develop data-driven decision-support tools for non-regulated diseases while providing a framework for their implementation [18]. Developing a cross-country tool that overcomes above mentioned challenges requires expertise in diagnostics and large-scale data processing, as well as social sciences to ensure that stakeholders’ needs are fully incorporated [19]. Within the DECIDE project, this essential combination of expertise enabled the development of the Cattle Barometer: an interactive dashboard tailored to the needs of farmers and veterinary professionals. The dashboard integrates information on respiratory pathogens, production systems, sampling strategies, and diagnostic analyses from multiple laboratories across Europe. The objective of this work is to present the development of the Cattle Barometer and its initial evaluation in practice.

## Methods

### Study context

This tool was developed as part of the DECIDE project (Horizon2020, No. 101000494, www.decideproject.eu), which aimed to develop data-driven support tools to aid with disease control and management in cattle, pigs, poultry, and salmon [18]. The development of the Cattle Barometer was supported by a multidisciplinary team of experts in BRD, data processing, and social sciences. Through iterative collaboration, the team continuously refined the tool, with all components related to data processing (e.g. ontologies) being made open access to enable future adaptation to other species and syndromes. The social science team employed a living-lab approach in which multiple rounds of end-user evaluation of the Cattle Barometer were conducted. These evaluation data are not explicitly included in the present paper but were integrated into the development process and are documented elsewhere [16, 17].

### Diagnostic data acquisition

Laboratories in Belgium, The Netherlands, Ireland, and France were contacted and asked to provide anonymised diagnostic test results from cattle with respiratory tract problems under a data transfer agreement. Data was collated by the labs according to the data script provided by Ghent University, exported to a CSV or Excel file format and sent to Ghent University. The data could consist of (several) individual samples or pooled samples, and included a anonymised farm identification number, date of sampling, identified pathogen (e.g. bovine respiratory syncytial virus, parainfluenza type-3, *M. bovis*, *Mannheimia haemolytica*, .), sample type (e.g. broncho alveolar lavage, swab, .), method of pathogen identification (PCR, nanopore sequencing or culture), and a proxy for location (municipality, province, .). The spatial precision of the location proxy was determined by each laboratory, depending on local farm density. In Belgium, for example, using the municipality level still ensures adequate anonymisation. In other countries, a coarser resolution (e.g. the provincial level), may have been required to maintain anonymity.

### Data cleaning and wrangling

The purpose of the data processing was to prepare the dataset for visualization of trends in sample positivity rates of specific pathogens at the farm level. As the raw data was heterogenous (across different languages, formats, and diagnostic tests), data cleaning and homogenisation was necessary. Scripts for processing the data were first created in R (Version 4.1.1) for each lab. Subsequently, scripts were also generated in Python (Version 3.11) and Stata (version 17). The data processing consisted of data import, cleaning, filtering, anonymisation, reorganisation, standardisation, and aggregation. Codes can be consulted here: https://github.com/decide-project-eu/cattle-use-case-barometer/.

In short, data for active monitoring or collected as part of research projects were excluded when indicated in the datasets. Only those with a diagnostic purpose in case of respiratory symptoms or outbreaks and analysed by PCR, nanopore sequencing or culture were included in the final dataset. An extra anonymisation step was added by applying SHA-256 hashing in both R (sha256_hash) and Python (hashlib.sha256) to privacy sensitive data such as farm identifiers.

### Interpretation of test results (standardisation)

Laboratory data from individual animals were harmonized by converting descriptive (e.g. ‘positive’, ‘present’) and semi-quantitative results (e.g. +, +++) into a binary outcome, with 1 indicating pathogen presence, and 0 indicating pathogen absence. Data with missing test results or dubious PCR test results, such as ‘not conclusive’ and ‘no test results due to inhibition’ were classified as ‘not analysed’ (NA). Culture results were expected to be either positive (1) or negative (0) for *Pasteurellaceae*. For example, when a sample (e.g. BAL) has been cultured on blood agar plates, we assumed that the sample was tested for *P. multocida* (PM), *M. haemolytica* (MH), and *Histophilus somni* (HS) simultaneously. When only *P. multocida* is present, the result for that specific sample is reported as: PM (1), MH (0), HS (0). Dubious test results due to overgrowth by for example *Proteus spp.* were interpreted as ‘not analysed’ (NA) for all *Pasteurellaceae*. Whether a result was considered test-positive or test-negative in case of nanopore sequencing, was at the discretion of the laboratory (cutoff values may differ per lab).

### Final dataset at herd level (aggregation)

To prepare the data for aggregation to farm status, all dates were set to a floored date, being the first of the month in which the sample was taken. Subsequently, the data were aggregated to the farm status per month and year, based on the floored date and anonymized farm ID. When at least one test from an individual animal was positive for a specific pathogen, the farm was considered positive for that pathogen in the corresponding month and year, irrespective of the presence of other pathogens. Co-pathogen combinations were not evaluated separately. The underlying dataset per laboratorium consisted of anonymous herd-level records including floored sampling date, pathogen (Bovine respiratory syncytial virus [BRSV], Bovine corona virus [BCV], Parainfluenza type-3 virus [PI3], PM, MH, HS, *M. bovis*), breed or production type (beef, dairy, mixed, veal, suckler), sample type (autopsy, swab, bronchoalveolar lavage [BAL], transtracheal aspirate or wash [TTA]), diagnostic test (culture, PCR, nanopore sequencing), laboratory reference, and country or province of origin. In the absence of a proxy for location, samples were allocated to the country.

Finally datasets from different labs were combined into one database, which subsequently could be connected to the visualisation tool (Table 1).

**Table 1:** Final standardised and anonymised data format after processing laboratory submission

Lab_reference	Country	Breed	Floored_date	Province	Farm_ID	Diagnostic_test	Sample_type	Pathogen	Result
3	Belgium	Beef	1-1-2020	East Flanders	2f93bf405d960b4b9952b3b71cdd6f628c0e3c948db1645e522092635c73deb1	Culture	BAL	BCV	NA
3	Belgium	Beef	1-1-2020	East Flanders	2f93bf405d960b4b9952b3b71cdd6f628c0e3c948db1645e522092635c73deb1	Culture	BAL	BRSV	NA
3	Belgium	Beef	1-1-2020	East Flanders	2f93bf405d960b4b9952b3b71cdd6f628c0e3c948db1645e522092635c73deb1	Culture	BAL	HS	0
3	Belgium	Beef	1-1-2020	East Flanders	2f93bf405d960b4b9952b3b71cdd6f628c0e3c948db1645e522092635c73deb1	Culture	BAL	MB	0
3	Belgium	Beef	1-1-2020	East Flanders	2f93bf405d960b4b9952b3b71cdd6f628c0e3c948db1645e522092635c73deb1	Culture	BAL	MH	0
3	Belgium	Beef	1-1-2020	East Flanders	2f93bf405d960b4b9952b3b71cdd6f628c0e3c948db1645e522092635c73deb1	Culture	BAL	PI3	NA
3	Belgium	Beef	1-1-2020	East Flanders	2f93bf405d960b4b9952b3b71cdd6f628c0e3c948db1645e522092635c73deb1	Culture	BAL	PM	0
3	Belgium	Beef	1-1-2020	East Flanders	2f93bf405d960b4b9952b3b71cdd6f628c0e3c948db1645e522092635c73deb1	PCR	BAL	BCV	1
3	Belgium	Beef	1-1-2020	East Flanders	2f93bf405d960b4b9952b3b71cdd6f628c0e3c948db1645e522092635c73deb1	PCR	BAL	BRSV	1
3	Belgium	Beef	1-1-2020	East Flanders	2f93bf405d960b4b9952b3b71cdd6f628c0e3c948db1645e522092635c73deb1	PCR	BAL	HS	0
3	Belgium	Beef	1-1-2020	East Flanders	2f93bf405d960b4b9952b3b71cdd6f628c0e3c948db1645e522092635c73deb1	PCR	BAL	MB	1
3	Belgium	Beef	1-1-2020	East Flanders	2f93bf405d960b4b9952b3b71cdd6f628c0e3c948db1645e522092635c73deb1	PCR	BAL	MH	0
3	Belgium	Beef	1-1-2020	East Flanders	2f93bf405d960b4b9952b3b71cdd6f628c0e3c948db1645e522092635c73deb1	PCR	BAL	PI3	1
3	Belgium	Beef	1-1-2020	East Flanders	2f93bf405d960b4b9952b3b71cdd6f628c0e3c948db1645e522092635c73deb1	PCR	BAL	PM	NA

*BCV*  Bovine coronavirus, *BRSV*  Bovine respiratory syncytial virus, *PI3*  Parainfluenza type-3, *HS*  Histophilus somni, *MB*  Mycoplasma bovis, *MH*  Mannheimia haemolytica, *PM*  Pasteurella multocida

To address data heterogeneity in a scalable way, a species-specific ontology was developed within the Livestock Health Ontology (LHO) framework. Ontologies provide formal, explicit representations of concepts and relationships within a domain, thereby supporting interoperability, semantic integration, and advanced querying [20]. The LHO, available on BioPortal (https://bioportal.bioontology.org/ontologies/LHO), provides a standardised structure for cattle health concepts and enables datasets from different laboratories to be mapped into a uniform semantic layer.

### Development of the interactive dashboard

The interactive dashboard was developed in Tableau Desktop (www.tableau.com) and consists of 7 worksheets to enable multidimensional exploration of the diagnostic laboratory data. Continuous date fields were retained to allow different levels of temporal aggregation and filtering. Several calculated fields were created within Tableau to support standardised summaries across visualisations. These included the total number of samples, number of positive results, the proportion of positives, and a unified measure (count or percentage) enabling flexible chart construction across different worksheets. Aggregation functions were applied to compute temporal or geographical summaries. For maps, geographic variables (country and/or province) were linked to automatically generated spatial coordinates. To support real-time subsetting and comparative analysis, a comprehensive set of interactive filters was implemented. Action filters were used to synchronise views: selecting a category in one chart automatically updates all other worksheets. This design permits dynamic exploration of relationships between pathogens, production systems, diagnostic test types, sampling context, and geographic distribution.

Throughout dashboard development, calculated fields and aggregated outputs were cross-validated against manually generated summaries and pivot tables derived from the raw dataset. Visual components were repeatedly tested with different filter combinations to ensure internal consistency and correct propagation of action filters. The final Tableau workbook can be downloaded (https://decide-project-eu.github.io/case-studies-website/case-studies/cattle-barometer.html).

### First evaluation of the usability of the Cattle Barometer

#### Survey design and distribution

To evaluate the usability of the Cattle Barometer, a survey was designed in French, Dutch, and English on the Ballpark platform (https://ballparkhq.com/). Invitations to participate were distributed to veterinarians, farmers, laboratory scientists and advisors in Ireland, the Netherlands, Belgium, France, and the United Kingdom via email, through a QR code displayed during presentations or provided upon request. An invitation to complete the survey was sent via email to veterinarians in Ireland (*n* = 18) and the UK (*n* = 25) who had previously completed a survey on technology use on beef and dairy farms and were interested in taking part in further research [16]. An invitation to complete the survey was sent via email to six veterinarians in Belgium based on their potential interest in using epidemiological data for both professional and research purposes. In the Netherlands, a QR code linking to the survey was presented during a lecture on respiratory infection in calves given at a meeting for veterinary professionals at the University of Utrecht. An invitation to complete the survey was also sent via email to veterinarians in the Netherlands (*n* = 25) that were classified as potential end users of the cattle dashboard. Finally, during a calf health day in the Netherlands, participating veterinarians were presented the dashboard and received the survey upon request.

The survey was open between October 2024 and March 2025. No compensation was provided for participation in the survey. The target sample size for the usability survey was 16 ± 4 respondents, as this is the number required to identify major, moderate, minor and cosmetic problems [21].

Participants first completed questions regarding their demographic and professional characteristics. They then explored the prototype tool while their screen activity and audio were recorded, after which they provided written initial feedback. To further assess usability in practice, participants were assigned a task requiring them to identify the percentage of positive samples in Ireland in December 2022.

The next part of the evaluation consisted of the System Usability Scale (SUS). This is a standardised questionnaire used to assess the perceived usability of a system, such as a dashboard, within its context of use (Table 3). The SUS consists of ten alternating statements, where odd numbered statements are positively phrased and even numbered statements are negatively phrased (Table 3). The respondent rates each statement using a 5-point Likert scale, from strongly disagree to strongly agree. Therefore, each respondent gives a value between 1 and 5 for each SUS statement, where 1 represents strongly disagree and 5 represents strongly agree. It provides a single usability score on a 0–100 scale, enabling benchmarking across products or against industry averages [22]. Because it consists of ten statements, it is quick to complete and offers a rapid overview of usability. The SUS has been validated in French, Dutch, and English [23, 24], and has been shown to produce reliable results, even in small sample sizes [25–27]. In addition, participants were asked whether they would use the tool in the future and to elaborate on their reasoning in open-ended responses, including suggestions for improvement. Finally, attitudes toward data sharing were assessed by asking participants to rate three statements concerning their comfort with making the tool’s outputs available to veterinarians, farmers, and the general public.

#### Survey analysis

The survey data were exported to an Excel file and Dutch and French language were translated into English for analysis. Descriptive statistical analyses were carried out in StataNow 18. The SUS score was calculated as follows: (1) for each of the odd numbered statements, subtract 1 from their value; (2) for each of the even numbered statements, subtract their value from 5; (3) sum the 10 adjusted scores; and (4) multiply the total by 2.5. This results in a score of up to 100, where a score of 68 is often considered a benchmark for average usability [28].

The participants’ responses to the open-ended survey questions and the video recordings of participants trialing the Cattle Barometer were included in the qualitative data analysis. The video recordings were downloaded from the Ballpark software, and each recording was watched by CD, who noted down any usability issues identified. The notes and qualitative survey data were imported into NVivo to aid qualitative analysis. The data were coded against four categories: content, design, navigation, and terminology. These categories were chosen a priori based on established research that identified content clarity, interface design, navigational structure, and terminology as discrete dimensions influencing user experience [29]. In each of these categories, sub-categories were inductively developed from the data. Usability issues were additionally qualitatively classified into high, moderate, and low severity based on their observed impact on users’ ability to understand content and complete tasks. For example, if the usability issue made it not possible to complete the task for some participants, then this was classed as a high severity issue. If the usability issue made it difficult to complete the task, but the users did complete the task after additional time, this was classed as a moderate severity issue. If the usability issue created a small inconvenience when carrying out the task, and few users reported the issue, then this was classed as a minor severity issue.

## Results

### Descriptives of Cattle Barometer data

Between 2022 and 2024, 14 datasets were received from 6 different diagnostic laboratories across 4 countries. The data consisted of all submitted individual and pooled BRD samples from 2016 until 2024. After aggregation, the final dataset included samples from 8432 farms (4804 from Belgium, 1179 from the Netherlands, 799 from Ireland, and 1650 from France).

This covers roughly 32.2%, 7.2%, 0.8%, and 1.4% of the Belgian, Dutch, Irish and French farms, respectively [30–33]. Beside demographic information about the origin of the samples, the data consisted of test results of BRSV (*n* = 15287), BCV (*n* = 15271), PI3 (*n* = 15290), *M. bovis* (*n* = 15726), MH (*n* = 17448), PM (*n* = 17447) and/or HS (*n* = 17464).

### Functional architecture of the dashboard

**Fig. 1:**
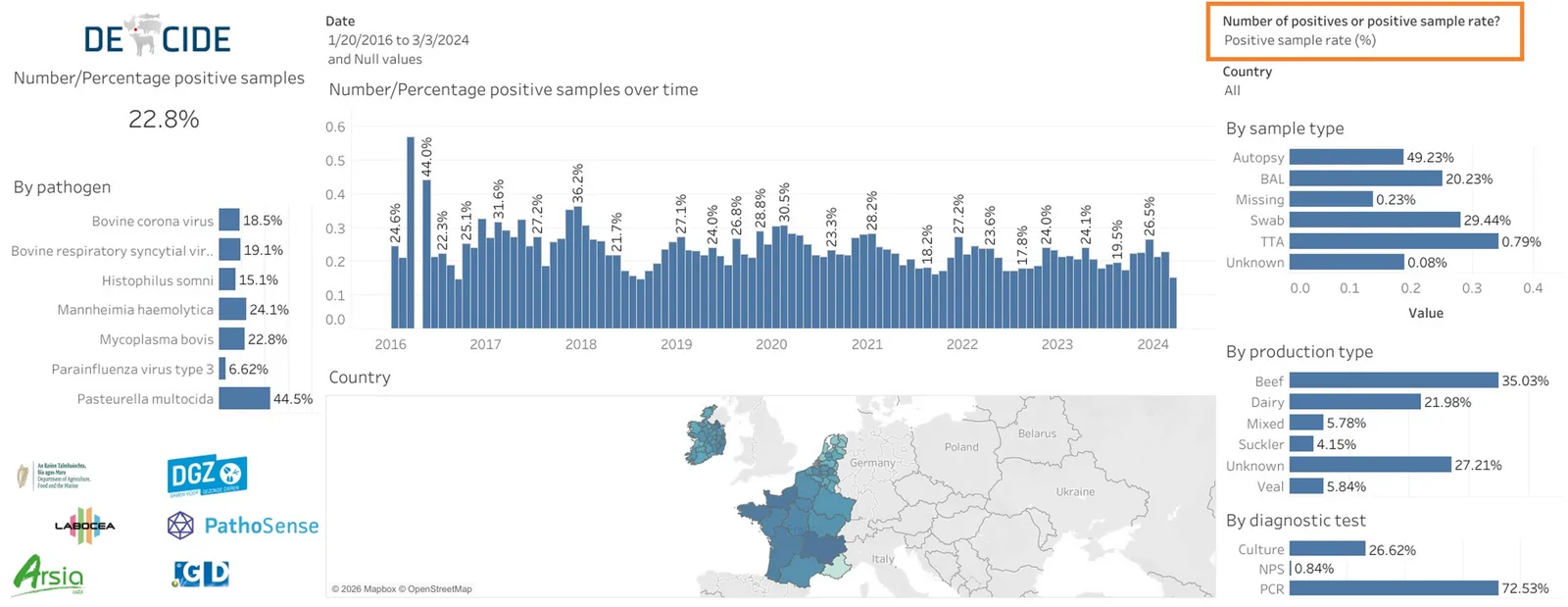
The default display of the number/percentage positive samples over time in the interactive dashboard ‘The Cattle Barometer’, including data from 6 laboratories over 4 countries. The metric toggle in the upper right corner (square, orange) allows users to switch between displaying the proportion of tested farms that were positive (“percentage”) and the absolute number of positive farms (“count”)

The first version of the Cattle Barometer presents an interactive visualisation of laboratory-confirmed respiratory pathogens in cattle across participating European countries (Fig. 1).

**Fig. 2:**
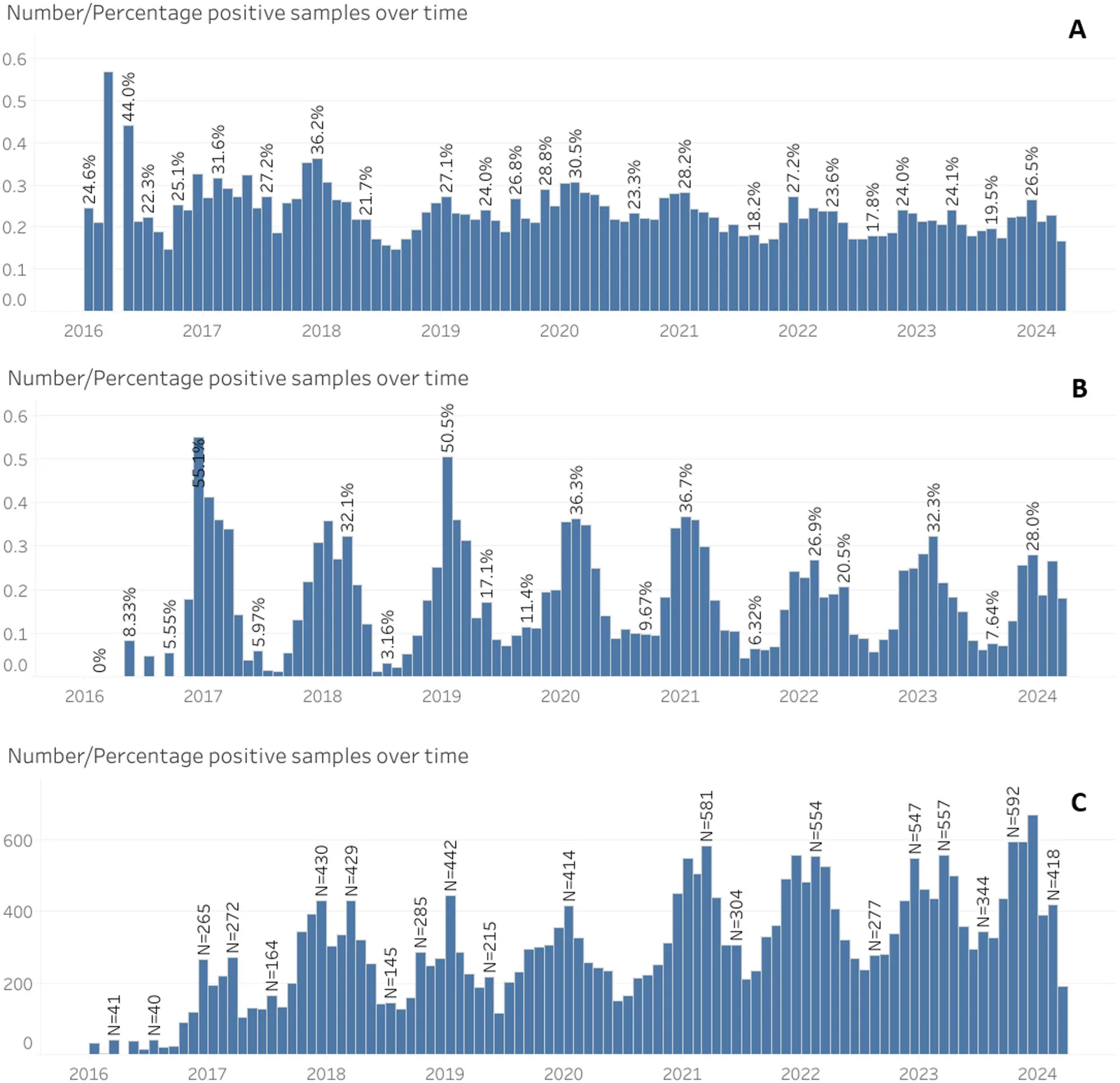
**A** The default display of the number/percentage positive samples over time in the interactive dashboard ‘Cattle Barometer’. The chart displays the monthly percentage of samples testing positive for all pathogens together. A continuous date slider above the graph enabled restriction to any defined temporal window. **B** Monthly variation in the proportion of BRSV-positive samples between 2016 and 2024, showing recurrent seasonal peaks in the proportion of positive samples. **C** Monthly count of diagnostic testing for all pathogens from 2016 to 2024, showing marked fluctuations in testing intensity over time

The central element of the dashboard is a time series plot that is able to show the number/percentage of positive samples per pathogen at the farm level. By default, the chart displays the monthly percentage of samples testing positive for all pathogens together. A continuous date slider above the graph enabled restriction to any defined temporal window (Fig. 2A). A separate map view plotted country-level or province-level metrics using generated longitude and latitude fields, allowing visual inspection of potential spatial patterns (e.g. colour gradient) (Fig. 1). By hoovering over the bars or map both count and percentage per month or location are shown.

The metric toggle allows users to switch between displaying the proportion of tested farms that were positive (“percentage”) and the absolute number of positive farms (“count”). This toggle, located in the upper right corner, dynamically recalculates the values to either count or percentage (Fig. 1, orange square). The percentage metric contextualises pathogen detections relative to testing intensity, enabling users to distinguish between increases driven by higher diagnostic submission rates and those reflecting a true rise in positivity, for example seasonal peaks of BRSV (Fig. 2B). Conversely, the count metric is useful for visualising absolute diagnostic burden and apparent seasonal peaks, such as increased sampling in the winter time (Fig. 2C).

**Fig. 3:**
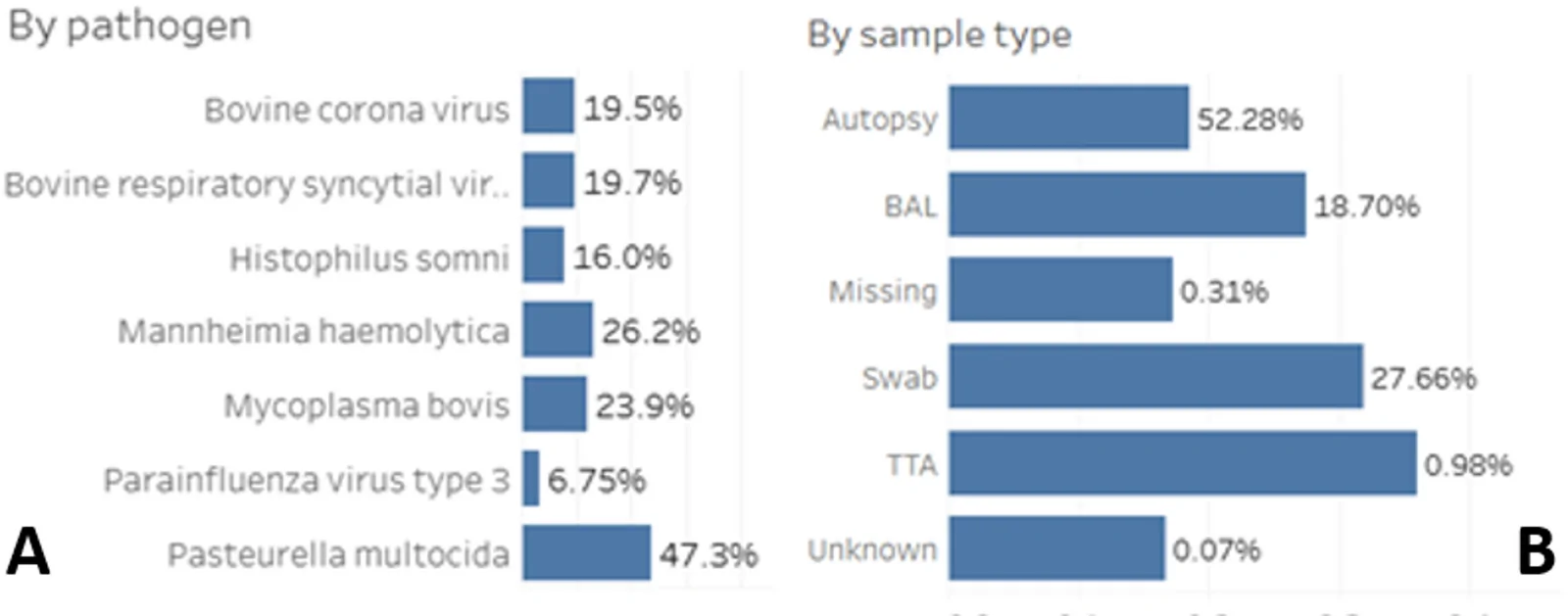
**A** Pathogen-level filter displaying the percentage of positive samples for each detected pathogen. Could also be shown as count of positive samples. **B** Sample type filter summarising positive test results by sample type (percentage or count)

A categorical selector on the left side of the dashboard allows users to choose a specific pathogen (Fig. 3A), triggering the automatic highlighting of the corresponding line in the time series and the suppression or de-emphasis of others. The dashboard includes additional filters, such as sample type (Fig. 3B), production type, and diagnostic test. Applying a filter dynamically recalculates the underlying dataset and refreshes the visualisation in real time. This enables users to explore spatial or methodological subsets of the data and observe how trends differ across contexts. Selecting a pathogen in the dashboard adjusts the visualisation, and activates an informational panel that provides pathogen-specific background. The dashboard can be explored here: https://decide-project-eu.github.io/case-studies-website/tutorials/cattle-barometer.html.

### Feedback on first version Cattle Barometer

Twenty-five participants evaluated the usability of the Cattle Barometer; their demographics are shown in Table 2. Out of the 25 participants, 15 (60%) successfully completed the provided task i.e. identify the percentage of positive samples in Ireland in December 2022, with a mean time of 158 s (SD: 121s, median: 132s). The distribution of answers to the SUS statements and availability of data is shown in Tables 3 and 4, respectively. The mean SUS score was 60 (SD: 19, median: 57.5), indicating that the usability score fell below the average benchmark of 68, which is still acceptable, but suggests room for improvement. In addition, 19 (76%) participants said they would use the tool in the future, and 6 (24%) said that they would not.

**Table 2:** Demographics of 25 participants in the Cattle barometer survey

Factor	Options	*N*	%
*Gender*	Female	13	52
	Male	12	48
*Age*	≤ 30	4	16
	31–40	10	40
	41–50	5	20
	51–60	5	20
	61–70	1	4
*Role* ^a^	Advisor	3	12
	Farmer	3	12
	Laboratory scientist	3	12
	Researcher	6	24
	Veterinarian	15	60
*Country*	Belgium	6	24
	France	7	28
	Ireland	4	16
	The Netherlands	5	20
	UK	3	12

^a﻿^Participants were able to choose more than 1 answer

**Table 3:** Participants’ ratings on the statements in the SUS for the Cattle Barometer

	Strongly disagree	Disagree	Neutral	Agree	Strongly agree	Mean
1: I think that I would like to use this tool frequently	4 (16%)	5 (20%)	10 (40%)	3 (12%)	3 (12%)	2.84
2: I found the tool unnecessarily complex	2 (8%)	9 (36%)	8 (32%)	6 (24%)	0 (0%)	2.72
3: I thought the tool was easy to use	2 (8%)	10 (40%)	7 (28%)	5 (20%)	1 (4%)	2.72
4: I think that I would need the support of a technical person to be able to use this tool	13 (52%)	6 (24%)	2 (8%)	2 (8%)	2 (8%)	1.96
5: I found the various functions in this tool were well integrated	2 (8%)	5 (20%)	6 (24%)	10 (40%)	2 (8%)	3.20
6: I thought there was too much inconsistency in this tool	8 (32%)	10 (40%)	3 (12%)	3 (12%)	1 (4%)	2.16
7: I would imagine that most people would learn to use this tool very quickly	1 (4%)	2 (8%)	6 (24%)	11 (44%)	5 (20%)	3.68
8: I found the tool very awkward to use	8 (32%)	5 (20%)	7 (28%)	3 (12%)	2 (8%)	2.44
9: I felt very confident using the tool	2 (8%)	10 (40%)	5 (20%)	6 (24%)	2 (8%)	2.84
10: I needed to learn a lot of things before I could get going with this tool	12 (48%)	4 (16%)	6 (24%)	1 (4%)	2 (8%)	2.08

**Table 4:** Participants’ answers on the availability of data in the Cattle Barometer

	Strongly disagree	Disagree	Neutral	Agree	Strongly agree	Mean
I am comfortable with the data outputs of this tool being available to vets.	0 (0%)	0 (0%)	3 (12%)	5 (20%)	17 (68%)	4.56
I am comfortable with the data outputs of this tool being available to farmers.	0 (0%)	4 (16%)	3 (12%)	3 (12%)	15 (60%)	4.16
I am comfortable with the data outputs of this tool being available to members of the public.	4 (16%)	3 (12%)	9 (36%)	2 (8%)	7 (28%)	3.20

The qualitative results from the open-ended questions and the microphone and screen analysis were categorised into design, navigation, content and terminology factors. For design, some participants commented on the overwhelming amount of data on the first page (severity: high), difficulties in controlling the map (the map zooms out very easily) (moderate), the small/unfitted size of the user interface (high), and the size or location of labels when hovering over a bar (low). For navigation, the lack of a ‘back’ or ‘return’ button (high) made navigating the filter function, especially the date filter, difficult and non-intuitive (high). Considering the content factors, the use of the tool appeared not to be very intuitive, as some participants suggested a video tutorial or a tutorial with fake data to practice with (moderate). Finally, on terminology, participants would rather see the date in European format (DD/MM/YYYY) instead of the current American format (MM/DD/YY) (moderate), and it should be adjusted to the language of their country (low). Also one user had some suggestions for the wording of titles (low).

When asked about the potential use of the tool, participants responded that the tool would not be that useful for individual farmers, but would be more useful for regional decision-making. For example, participant 5 (Farmer, Ireland) answered: *“As a farmer I’m not so sure. If I had a large problem with a particular outbreak*,* it would be good to know the prevalence within my country however*,* I feel it would be more useful to my vet or advisor to get a larger picture.”*, whereas participant 6 (Lab scientist and Vet, Belgium) would use the Cattle Barometer to: “*Verify the prevalence of respiratory pathogens in a certain region during a certain period.”*, participant 17 (Vet, UK) for: *“Just overall surveillance to see if disease patterns were changing overtime.”* and participant 14 (Researcher and Vet, France) to: “*Know the type of pathogens circulating in France and abroad*,* and the positivity rate.”*.

Some users suggested that the tool could support communication, such as by helping clinicians provide advice on disease prevention (participant 6, Lab scientist, Advisor and Vet, Belgium: “*This to help in the communication to farmers and vets on the importance of prevention and taking measures.”*). Users also thought that the tool could help guide research, for example, participant 17 (Vet, The Netherlands) wrote: “*As a reference/reference work for the situation in one’s own working area compared to other countries. For presentations. Perhaps also for collaboration within the participating countries if the data give reason to do so.”*, whereas participant 8 (Researcher, France) indicated: “*In the context of researching information for projects*”.

## Discussion

This study describes the development and initial user evaluation of the Cattle Barometer: a cross-country decision-support tool designed to enhance monitoring and control of respiratory disease in cattle. The BRD complex was selected as a proof of concept as it represents one of the most important non-regulated disease complexes in calves [1, 2]. The resulting dashboard functions as an exploratory analytic tool, enabling future users to explore pathogen detection patterns, seasonal and temporal trends, and changes in diagnostic submissions and proportion of positive samples. Its interactive Tableau interface allows analyses across pathogens, regions, diagnostic tests, and sample types, while the combination of count- and percentage-based visualisations facilitates interpretation of both absolute and relative number of positive sample trends, including comparisons between countries. The Cattle Barometer builds on the growing use of digital systems that transform routinely collected veterinary diagnostic data into interactive decision-support tools, such as the Veterinary Investigation Diagnosis Analysis dashboards [34], and the Ontario Interactive Animal Pathogen Dashboards [35]. In addition, international organisations (e.g. WOAH, EFSA) advocate interoperable laboratory data systems and harmonised infrastructures to strengthen disease detection and support rapid cross-border risk assessment [36, 37]. The Cattle Barometer contributes to these developments by demonstrating the feasibility of semantic interoperability between veterinary diagnostic laboratories from different countries through shared ontologies, and standardised data processing workflows.

Our main goal was to develop a tool for veterinarians to support farmer–veterinarian interaction, and for other stakeholders in the livestock industry to access actionable insights to optimise herd management practices and minimise disease-related losses. For example, veterinary professionals could use the Cattle Barometer to monitor disease trends in specific regions, make informed decisions about vaccination strategies, and allocate resources to high-risk areas or avoid purchase from regions with high proportion of positive samples. Whereas researchers and government epidemiologists could leverage it to identify patterns and contributing factors to disease outbreaks, facilitating the development of targeted interventions and prevention strategies at (inter)national level. The Cattle Barometer may also raise stakeholders’ awareness of the importance of taking samples for diagnostic purposes and show laboratories the potential to provide more context for individual diagnostic results.

The evaluation confirmed that the Cattle Barometer is promising but still requires refinement to achieve optimal user-friendliness. Prior work has shown that farmers and veterinarians adopt technological tools only when navigation is intuitive and information is easily accessible [16, 17]. This aligns with our survey results: participants valued clear visual outputs and straightforward interactions. However, we should consider that, although the majority of survey respondents (strongly) agreed to share data with their vets (88%) and farmers (72%), a fair number disagreed with being comfortable sharing data outputs with members of the public (28%). This is in line with a previous study showing that livestock producers have historically been cautious about sharing health-related business data, particularly when outputs may become visible beyond trusted professional networks [38, 39]. Future development will need to balance these considerations to maintain usability while ensuring appropriate data governance [40].

Almost half of the participants found the tool’s functions well integrated, and the majority (64%) said they anticipated learning to use the barometer quickly. Nonetheless, others found the tool not easy to use (48%) or inconsistent (16%), highlighting that although the basic structure is accessible, navigation and clarity can be improved. This also aligns with stakeholder suggestions for additional instructions or training materials to support the interpretation of pathogen trends and improve practical utility. As all stakeholders were included in the SUS, it may be interesting to separate farmers, veterinarians, and researchers to explore further needs within each target group [41]. Nevertheless, the SUS results and stakeholder perspectives provide clear direction for iterative improvements. Enhancing intuitiveness, strengthening guidance, and designing a balanced access model will be essential for ensuring that the Cattle Barometer is both acceptable and valuable for routine veterinary and farm-level decision-making.

A central challenge in developing the Cattle Barometer, consistent with previous studies exploring livestock data [9, 40], was the heterogeneity and interpretation of diagnostic data across laboratories. Differences in languages, formats, metadata completeness, diagnostic techniques, and sampling strategies, required substantial cleaning, homogenisation, and expert interpretation before integration. Despite these challenges, the collaboration among laboratories in Belgium, the Netherlands, Ireland, and France demonstrated the feasibility of cross-country integration. The tutorial created for participating labs supports this process by explaining data formatting requirements and linking to code resources (https://decide-project-eu.github.io/case-studies-website/tutorials/cattle-barometer.html).

Even though we were able to standardize the data, it is important to understand the underlying diagnostic data and its limitations when using the Cattle barometer. First of all, diagnostic submissions are not collected according to a standardised sampling protocol, as they are influenced by the clinical judgement of veterinarians, farmers’ willingness to submit samples, economic considerations, laboratory accessibility, and national diagnostic practices [39, 42–44]. Consequently, the sampled animals are unlikely to be representative of the cattle population within a region or country [44] This is also reflected in the estimated sampling coverage of farms in each country so far (1 to 32%). Therefore, the proportion of positive samples should not be interpreted as estimates of disease prevalence. The addition of a cattle density layer or heat map showing the geographical distribution of cattle populations could further improve interpretation of regional differences. Overlaying diagnostic results with livestock density would provide valuable epidemiological context and help users distinguish between areas with genuinely increased diagnostic activity and areas where high submission numbers primarily reflect larger cattle populations.

Secondly, because farms and laboratories were anonymised, submissions from the same farm to (multiple) participating laboratories could not be identified, potentially resulting in duplicate positive or negative herd records within a region. Finally, differences in diagnostic methods, including assays with varying sensitivities and specificities, further complicate comparisons between laboratories and countries [12, 45]. Greater transparency regarding diagnostic methods and test performance characteristics will be essential for improving the comparability and interpretation of future versions of the Cattle Barometer.

Taken together, these limitations emphasise that the Cattle Barometer should primarily be regarded as an exploratory monitoring tool for identifying temporal and spatial patterns in diagnostic activity and proportion of positive samples per pathogen, rather than as a direct measure of disease prevalence or incidence. Next to this, it could also be used the other way around: as a instrument that provides epidemiological context for laboratory findings.

The Cattle Barometer holds considerabel potential for extension beyond the BRD proof of concept. Opportunities for further development include universal coding of laboratory test names [14], expanding to additional species (e.g., pigs, poultry), adding other diagnostic tests (e.g. antibody ELISA), and incorporate more detailed clinical metadata (e.g., age, production stage, clinical signs). The design of species-specific ontology within the Livestock Health Ontology framework allows this expansion of the (Cattle) barometer, as demonstrated in ongoing open-acces initiatives within the DECIDE project (barometer for poultry and pigs) [46, 47]). Through this semantic foundation, the Cattle Barometer can evolve with minimal structural revision even as diagnostic technologies or pathogen panels expand. Integration with federated learning architectures or secure file-transfer pipelines could allow laboratories to contribute data without relinquishing ownership, thereby maintaining the principle that laboratories retain full control over their data [48]. During the DECIDE project this was developed, tested and deemed feasible for real-time data transfer between the laboratory in the Netherlands and Ghent University [49]. With continuous data updating, the Cattle Barometer could be used to develop epidemiological models for a more efficient diagnosis and prevention of BRD [50]. For example, seasonal increases in BRSV detections (Fig. 2B) may trigger early-warning alerts, enabling timely vaccination.

However, the success of the Cattle Barometer depends on sustained collaboration between laboratories. Nationally limited datasets risk the introduction of bias, as observed trends can reflect differences in region coverage, rather than actual disease occurrence. International integration of laboratory data (enabled by shared ontologies, federated acces, and harmonised workflows), reduces this bias by combining all available data rather than relying on partial datasets, thereby supporting accurate and representative monitoring. A concern for the future is that stakeholders may take scripts or workflows developed during the project, but stop contributing data, undermining the tool’s collective value. Maintaining a strong collaborative network is therefore essential for sustainable exploitation of the Cattle Barometer.

## Conclusions

The Cattle Barometer visualises pathogen-specific lab results of field samples obtained from cattle across Europe. It represents an important step toward harmonised, cross-country surveillance of respiratory pathogens. By integrating heterogeneous laboratory data through an ontology-driven pipeline and by considering usability and stakeholder needs, the tool lays the groundwork for early-warning systems, improved disease management, and enhanced awareness of BRD across Europe. Continued collaboration, further data enrichment, automisation, and expansion to additional species will be essential to realising its full potential.

## Data Availability

All materials, tools, and data products developed within the DECIDE framework will be made openly available in accordance with the project’s open-access policy.

## Abbreviations

BAL: Broncho-alveolar lavage
BCV: Bovine coronavirus
BRSV: Bovine respiratory syncytial virus
BRD: Bovine respiratory disease
HS: *Histophilus somni*
LHO: Livestock Health Ontology
LIMS: Laboratory Information Management System
MB: *Mycoplasma bovis*
MH: *Mannheimia haemolytica*
NA: Not analysed / Not available
PCR: Polymerase chain reaction
PI3: Parainfluenza type-3 virus
PM: *Pasteurella multocida*
SD: Standard deviation
SHA-256: Secure Hash Algorithm 256-bit
SUS: System Usability Scale
TTA: Transtracheal aspiration
